# Data on ^137^Cs concentration factor of freshwater fish and aquatic organisms in lake and river ecosystems

**DOI:** 10.1016/j.dib.2019.105043

**Published:** 2020-01-01

**Authors:** Yumiko Ishii, Shin-ichiro S. Matsuzaki, Seiji Hayashi

**Affiliations:** a Environmental Impact Assessment Section, Fukushima Branch, National Institute for Environmental Studies, 10-2 Fukasaku, Miharu Town, Tamura County, Fukushima, 963-7700, Japan; b Center for Environmental Biology and Ecosystem Studies, National Institute for Environmental Studies, 16–2 Onogawa, Tsukuba, Ibaraki, 305-8506, Japan

**Keywords:** Fukushima, Radiocesium, Aquatic biota, Concentration factor

## Abstract

This article provides the data which were analyzed in the research article “Different factors determine ^137^Cs concentration factors of freshwater fish and aquatic organisms in lake and river ecosystems” (Y. Ishii, S. S. Matsuzaki, S. Hayashi, 2019) [1]. Radionuclide accumulation in aquatic organism is defined in terms of the concentration factor (CF), which is calculated as the radionuclide concentration in the organism (Bq kg^−1^) divided by that in the surrounding water (Bq L^−1^). Quantification of the radionuclide CF allows estimation of environmental radionuclide transfer and the potential risks of consuming fish contaminated with the radionuclide. We calculated the ^137^Cs CF values for freshwater fish and aquatic organisms using the monitoring data of multiple sites in five rivers and three lakes of Fukushima in years 2013–2017 after the Fukushima Dai-ichi Nuclear Power Plant accident. The data also include the ^137^Cs activity concentration of the water and water chemistry data (pH, biochemical oxygen demand, chemical oxygen demand, dissolved oxygen, electric conductivity, salinity, total organic carbon, suspended solid concentration, turbidity) at each sampling location associated with each CF value.

**Table:** Specifications table

Subject area	*Environmental Science*
More specific subject area	*Pollution*
Type of data	*Tables and Figures*
How data was acquired	*Coaxial germanium detectors (Canberra GC2020 [Mirion Technologies, San Ramon, CA, USA], Canberra GC4020 [Mirion Technologies], ORTEC GMX 60–83 [Ametek Ortec, Oak Ridge, TN, USA], and GEM 40–76 [Ametek Ortec]) and well-type germanium detectors (Canberra GCW2523 [Mirion Technologies] and ORTEC GWL–90–15–XLB–AWT [Ametek Ortec])*
Data format	*Raw and analyzed*
Experimental factors	*The ^137^Cs activity concentrations were measured for the wet whole-body samples of freshwater fish and other aquatic organisms from five rivers and three lakes in Fukushima Prefecture after the Fukushima Daiich Nuclear Power Plant accident.*
Experimental features	*The ^137^Cs activity concentrations and their concentration factors, which are defined as the ratio of ^137^Cs activity in the samples (Bq kg^−1^ fresh weight) to that of ^137^Cs activity in the water (Bq L^−1^), were calculated for freshwater fish and other aquatics organisms.*
Data source location	*Uda river, Mano river, Niida river, Ota river, Abukuma river, Lake Hayama, Lake Akimoto, and Lake Inawashiro in Fukushima Prefecture, Japan*
Data accessibility	*Data presented in this article*
Related research article	[If your data article is related to a research article, please cite your associated research article here]. Author's name: Yumiko Ishii, Shin-ichiro S. Matsuzaki, Seiji Hayashi Title: Different factors determine ^137^Cs concentration factors of freshwater fish and aquatic organisms in lake and river ecosystems Journal: Journal of Environmental Radioactivity DOI: doi.org/10.1016/j.jenvrad.2019.106102

**Table:** 

**Value of the data** • This data can be useful for researchers for the estimation of the radiological risks associated with the freshwater ecosystem. • The data can be useful for comparison of ^137^Cs bioaccumulation with other regions or other ecosystems. • The data can be useful for further research of the factors affecting ^137^Cs accumulation in freshwater aquatic organisms.

## 1 Data

**Fig. 1:**
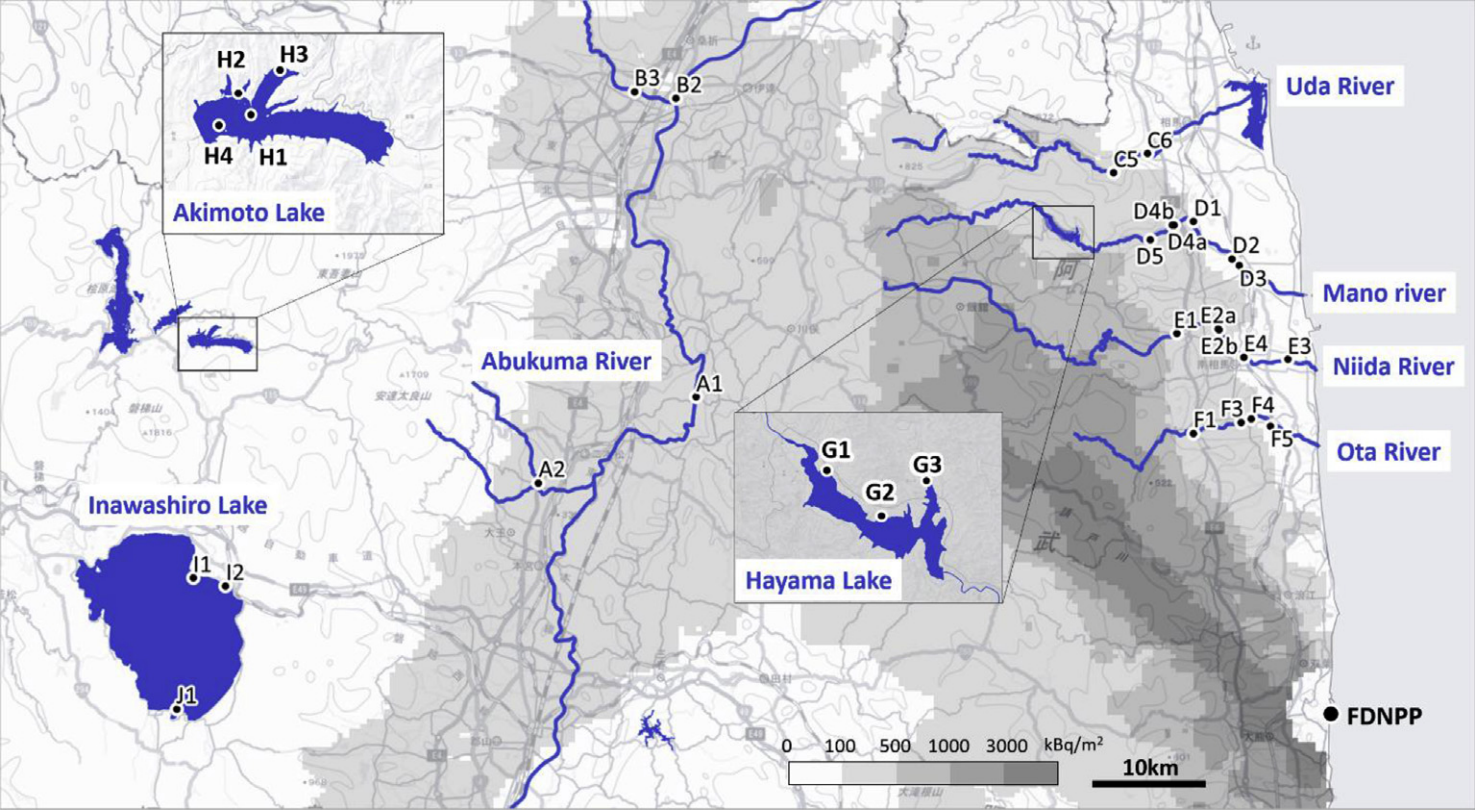
Map of locations of sampling sites. ^134^Cs and ^137^Cs total depositions in Fukushima are shown in gray scale, according to data from the 5th Airborne Radiation Monitoring by the Ministry of Education, Culture, Sports, Science, and Technology (MEXT) in 2012. This map used the digital topographic tile of the Geospatial Information Authority of Japan.

**Fig. 2:**
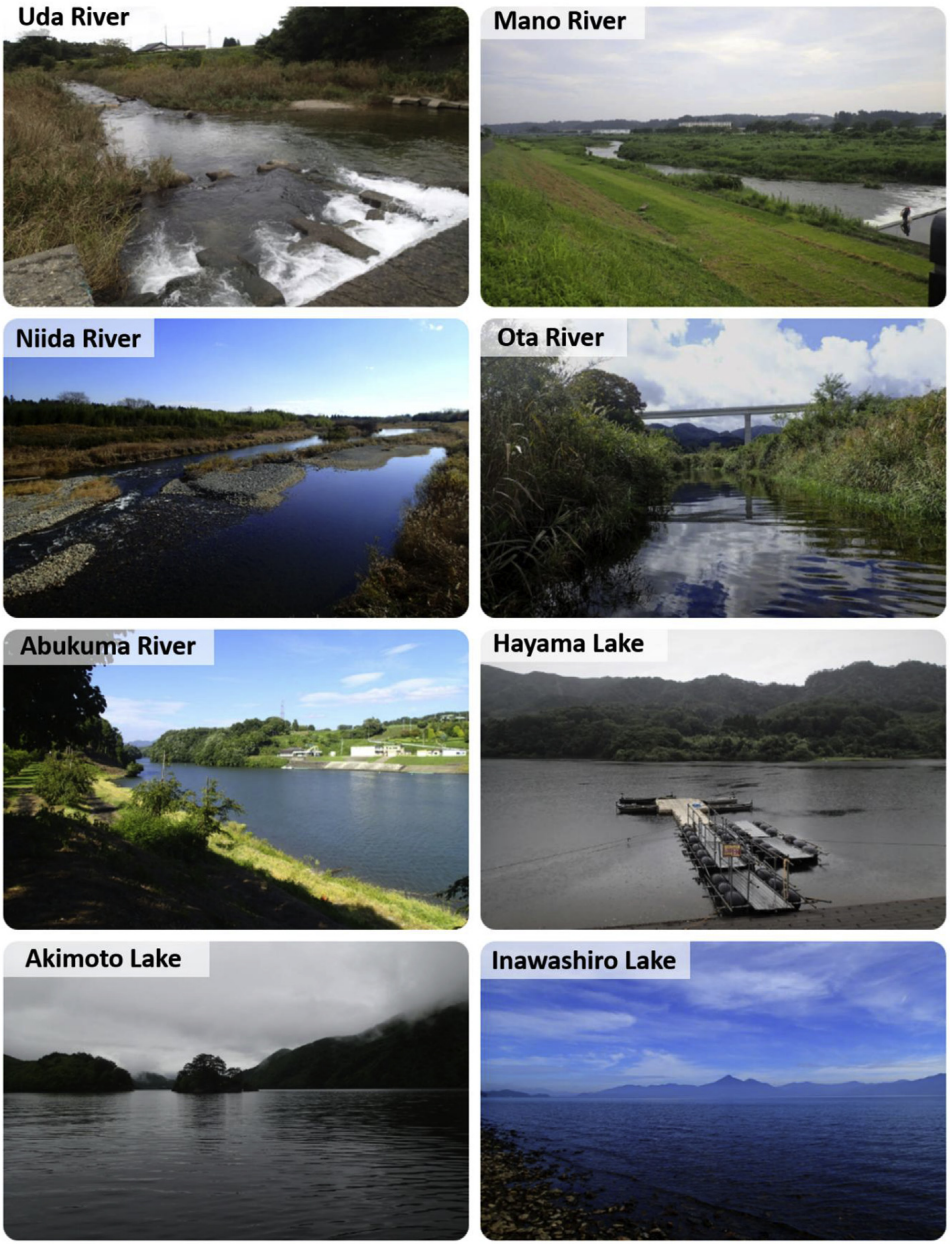
Pictures of the sampling sites.

The dataset contains the ^137^Cs activity concentrations and their concentration factors (CF), which are defined as the ratio of ^137^Cs activity in the samples (Bq.kg^−1^ fresh weight) to that of ^137^Cs activity in the water (Bq.L^−1^), of freshwater fish and other aquatic organisms in Fukushima. We compiled the data from the Radioactive Material Monitoring Surveys of the Water Environment [2] sponsored by the Japanese Ministry of the Environment (MOE). Information about the sampling sites in Fukushima is presented in Fig. 1 (Map of the sampling sites), Fig. 2 (Pictures of the sampling sites) and Table 1 (List of sampling sites). The ^137^Cs CF values are summarized for freshwater fish species (Table 2) and other freshwater aquatic organisms (Table 3). The datasets which were used to calculate the ^137^Cs CF values and associated water chemistries are attached as a supplementary file to this article as Appendix A (freshwater fish species) and Appendix B (other freshwater aquatic organisms).

**Table 1:** Latitude and longitude of sampling sites.

Site	Site code	Sampling point	Latitude (N)	Longitude (E)
Uda River	RU	C5	37.7644	140.8603
		C6	37.7765	140.8876
Mano River	RM	D1	37.7332	140.9254
		D2	37.7093	140.9565
		D3	37.7050	140.9622
		D4a	37.7309	140.9079
		D4b	37.7311	140.9096
		D5	37.7217	140.8899
Niida River	RN	E1	37.6614	140.9115
		E2a	37.6644	140.9453
		E2b	37.6641	140.9459
		E3	37.6447	141.0013
		E4	37.6463	140.9658
Ota River	RO	F1	37.5975	140.9250
		F3	37.6045	140.9637
		F4	37.6069	140.9720
		F5	37.6022	140.9874
Abukuma River	RA-A	A1	37.6206	140.5220
		A2	37.5657	140.3943
	RA-B	B2	37.8120	140.5058
		B3	37.8164	140.4719
Lake Hayama	LH	G1	37.7321	140.8127
		G2	37.7267	140.8223
		G3	37.7302	140.8307
Lake Akimoto	LA	H1	37.6575	140.1264
		H2	37.6616	140.1226
		H3	37.6653	140.1329
		H4	37.6551	140.1181
Lake Inawashiro	LI	I1	37.5047	140.1143
		I2	37.4995	140.1409
		J1	37.4203	140.1008

**Table 2:** ^137^Cs concentration factors (Lkg^−1^) for freshwater fish. AM: arithmetic mean, ASD: arithmetic standard deviation, GM: geometric mean, GSD: geometric standard deviation. For *Carassius* sp., *Cottus* sp., Loach, and *Rhinogobius* sp., multiple species were merged to a group. The merged species name and the number of samples for each species are listed in the “merged species name” column.

Fish species	Merged species name	Functional feeding group	Habitat	Ecosystem type	N	AM	ASD	GM	GSD	Min.	Max.
*Acheilognathus melanogaster*		Omnivore	Benthopelagic	River	3	9.8 × 10^2^	8.4 × 10^2^	7.6 × 10^2^	2.4	3.5 × 10^2^	1.9 × 10^3^
*Anguilla japonica* (Japanese eel)		Piscivore	Demersal	River	29	2.7 × 10^2^	2.6 × 10^3^	1.7 × 10^2^	3.0	6.5 × 10^0^	1.3 × 10^4^
*Candidia temminckii*		Omnivore	Benthopelagic	River	47	6.2 × 10^2^	4.1 × 10^2^	4.9 × 10^2^	2.0	7.1 × 10^0^	1.7 × 10^3^
Carassius sp.	*Carassius auratus langsdorfii* (67)*, Carassius cuvieri* (2)*, Carassius* sp. (27)	Omnivore	Benthopelagic	Lake	73	2.1 × 10^3^	1.2 × 10^3^	1.7 × 10^3^	2.1	2.3 × 10^2^	6.6 × 10^3^
				River	23	1.2 × 10^3^	7.4 × 10^2^	1.0 × 10^3^	2.2	1.1 × 10^2^	3.2 × 10^3^
*Channa argus*		Piscivore	Benthopelagic	Lake	1	3.1 × 10^3^	–	–	–	–	–
Cottus sp.	*Cottus pollux* (21)*, Cottus reinii* (5)	Omnivore	Demersal	Lake	2	1.0 × 10^3^	3.9 × 10^2^	9.8 × 10^2^	1.5	7.4 × 10^2^	1.3 × 10^3^
				River	24	1.0 × 10^3^	1.1 × 10^3^	6.5 × 10^2^	2.9	6.8 × 10^1^	3.8 × 10^3^
*Cyprinus carpio* (Common carp)		Omnivore	Benthopelagic	Lake	18	1.8 × 10^3^	7.0 × 10^2^	1.7 × 10^3^	1.5	8.7 × 10^2^	3.2 × 10^3^
				River	22	1.3 × 10^3^	1.5 × 10^3^	8.1 × 10^2^	2.7	9.6 × 10^1^	6.6 × 10^3^
*Gnathopogon elongatus*		Omnivore	Benthopelagic	River	11	1.1 × 10^3^	6.7 × 10^2^	7.9 × 10^2^	2.9	6.7 × 10^1^	2.1 × 10^3^
*Gymnogobius urotaenia*		Omnivore	Demersal	Lake	1	1.8 × 10^3^	–	–	–	–	–
				River	5	8.8 × 10^2^	5.3 × 10^2^	6.8 × 10^2^	2.5	1.6 × 10^2^	1.3 × 10^3^
*Hemibarbus barbus* (Japanese barbel)		Omnivore	Benthopelagic	Lake	46	2.2 × 10^3^	1.0 × 10^3^	1.9 × 10^3^	1.8	3.3 × 10^2^	4.7 × 10^3^
				River	29	8.2 × 10^2^	9.0 × 10^2^	4.5 × 10^2^	3.4	3.9 × 10^1^	4.1 × 10^3^
*Hypomesus nipponensis* (Pond smelt)		Planktivore	Pelagic	Lake	22	1.1 × 10^3^	5.9 × 10^2^	9.7 × 10^2^	1.6	3.1 × 10^2^	3.0 × 10^3^
				River	1	3.1 × 10^2^	–	–	–	–	–
*Ictalurus punctatus* (Channel catfish)		Piscivore	Demersal	River	13	5.6 × 10^2^	3.9 × 10^2^	4.1 × 10^2^	2.4	6.9 × 10^1^	1.1 × 10^3^
*Lepomis macrochirus* (Bluegill)		Omnivore	Benthopelagic	Lake	10	1.5 × 10^3^	7.0 × 10^2^	1.3 × 10^3^	1.7	4.1 × 10^2^	2.9 × 10^3^
				River	5	1.8 × 10^3^	2.9 × 10^3^	7.4 × 10^2^	4.5	1.7 × 10^2^	7.0 × 10^3^
Loach	*Misgurnus anguillicaudatus* (55)*, Cobitis biwae* (19)*, Noemacheilus barbatulus* (10)*, Nemacheilus toni* (5)*, Lefua echigonia* (3)	Omnivore	Demersal	Lake	2	1.9 × 10^2^	1.5 × 10^2^	1.6 × 10^2^	2.5	8.4 × 10^1^	3.0 × 10^2^
				River	90	7.0 × 10^2^	6.8 × 10^2^	4.6 × 10^2^	2.5	4.9 × 10^1^	3.7 × 10^3^
*Micropterus dolomieu* (Smallmouth bass)		Piscivore	Benthopelagic	Lake	65	4.9 × 10^3^	4.1 × 10^3^	3.8 × 10^3^	2.0	4.2 × 10^2^	2.5 × 10^4^
				River	27	1.1 × 10^3^	1.2 × 10^3^	6.3 × 10^2^	3.1	1.0 × 10^2^	5.8 × 10^3^
*Micropterus salmoides* (Largemouth bass)		Piscivore	Benthopelagic	Lake	9	4.3 × 10^3^	3.4 × 10^3^	3.1 × 10^3^	2.4	1.0 × 10^3^	1.0 × 10^4^
				River	3	1.1 × 10^3^	1.2 × 10^3^	7.0 × 10^2^	4.0	1.6 × 10^2^	2.6 × 10^3^
*Oncorhynchus masou* (Masu salmon)		Piscivore	Benthopelagic	Lake	33	2.7 × 10^3^	1.8 × 10^3^	2.1 × 10^3^	2.4	1.0 × 10^2^	8.6 × 10^3^
				River	62	1.0 × 10^3^	1.8 × 10^3^	5.3 × 10^2^	2.9	5.4 × 10^1^	9.8 × 10^3^
*Oncorhynchus mykiss* (Rainbow trout)		Piscivore	Benthopelagic	Lake	3	3.4 × 10^3^	3.5 × 10^3^	2.1 × 10^3^	3.8	5.2 × 10^2^	7.4 × 10^3^
*Opsariichthys platypus* (Pale chub)		Omnivore	Benthopelagic	Lake	13	9.6 × 10^2^	5.6 × 10^2^	8.4 × 10^2^	1.6	4.9 × 10^2^	2.3 × 10^3^
				River	75	8.8 × 10^2^	6.3 × 10^2^	6.4 × 10^2^	2.4	5.3 × 10^1^	2.8 × 10^3^
*Phoxinus lagowskii*		Omnivore	Benthopelagic	Lake	6	5.1 × 10^2^	2.9 × 10^2^	4.3 × 10^2^	1.9	1.5 × 10^2^	9.5 × 10^2^
				River	35	5.3 × 10^2^	4.5 × 10^2^	3.9 × 10^2^	2.3	8.0 × 10^1^	2.4 × 10^3^
*Plecoglossus altivelis* (Ayu)		Herbivore	Benthopelagic	River	59	1.1 × 10^3^	1.0 × 10^3^	7.4 × 10^2^	3.0	3.0 × 10^1^	5.8 × 10^3^
*Pseudobagrus tokiensis*		Omnivore	Demersal	River	8	5.0 × 10^2^	3.8 × 10^2^	4.0 × 10^2^	2.0	1.7 × 10^2^	1.3 × 10^3^
*Pseudogobio esocinus*		Omnivore	Benthopelagic	Lake	17	7.4 × 10^2^	2.3 × 10^2^	7.0 × 10^2^	1.4	3.5 × 10^2^	1.1 × 10^3^
				River	12	5.7 × 10^2^	2.6 × 10^2^	5.1 × 10^2^	1.6	1.8 × 10^2^	1.0 × 10^3^
*Pseudorasbora parva*		Omnivore	Benthopelagic	River	1	8.4 × 10^2^	–	–	–	–	–
Rhinogobius sp.	*Rhinogobius fluviatilis* (18), *Rhinogobius nagoyae* (7), *Rhinogobius kurodai* (1), *Rhinogobius* sp. (36)	Omnivore	Benthopelagic	River	62	2.3 × 10^3^	1.3 × 10^3^	1.9 × 10^2^	2.1	1.1 × 10^2^	5.7 × 10^3^
*Salvelinus leucomaenis* (Whitespotted char)		Piscivore	Benthopelagic	Lake	46	4.1 × 10^3^	1.8 × 10^3^	3.7 × 10^3^	1.6	1.1 × 10^3^	8.7 × 10^3^
				River	3	2.4 × 10^3^	2.8 × 10^3^	1.1 × 10^3^	5.6	1.8 × 10^2^	5.5 × 10^3^
*Sarcocheilichthys variegatus*		Omnivore	Benthopelagic	River	4	6.1 × 10^2^	3.6 × 10^2^	4.4 × 10^2^	3.1	8.2 × 10^1^	8.6 × 10^2^
*Silurus asotus* (Japanese catfish)		Piscivore	Demersal	Lake	17	6.4 × 10^3^	5.5 × 10^3^	3.9 × 10^3^	3.2	4.5 × 10^2^	1.8 × 10^4^
				River	23	3.0 × 10^3^	4.9 × 10^3^	1.7 × 10^3^	2.6	2.7 × 10^2^	2.4 × 10^4^
*Tribolodon hakonensis* (Japanese dace)		Omnivore	Benthopelagic	Lake	69	2.6 × 10^3^	1.2 × 10^3^	2.3 × 10^3^	1.7	3.7 × 10^2^	6.0 × 10^3^
				River	123	1.1 × 10^3^	8.2 × 10^2^	8.2 × 10^2^	2.6	4.6 × 10^1^	3.9 × 10^3^
Tridentiger brevispinis		Omnivore	Demersal	River	3	1.2 × 10^3^	2.6 × 10^2^	1.2 × 10^3^	1.2	1.0 × 10^3^	1.5 × 10^3^

**Table 3:** ^137^Cs concentration factors (Lkg^−1^) for freshwater aquatic organisms. Samples which were identified to the species level are listed in the species name column. AM: arithmetic mean, ASD: arithmetic standard deviation, GM: geometric mean, GSD: geometric standard deviation.

Aquatic organisms	Species name	Ecosystem type	N	AM	ASD	GM	GSD	Min.	Max.
Litter		River	112	3.2 × 10^3^	3.5 × 10^3^	1.9 × 10^3^	3.0	7.6 × 10^1^	2.4 × 10^4^
Plankton		Lake	40	1.7 × 10^3^	3.7 × 10^3^	4.1 × 10^2^	5.2	1.9 × 10^1^	2.0 × 10^4^
Periphyton		River	116	7.3 × 10^3^	5.4 × 10^3^	5.3 × 10^3^	2.4	2.7 × 10^2^	2.6 × 10^4^
Moss	*Sphagnum* sp.(7)	River	9	3.3 × 10^3^	3.0 × 10^3^	2.3 × 10^3^	2.6	4.9 × 10^2^	1.0 × 10^4^
Filamentous algae	*Spirogyra* sp.(14), *Oedogonium* sp. (2), *Cladophora* sp.(1)	River	15	8.2 × 10^2^	1.2 × 10^3^	3.2 × 10^2^	4.3	1.6 × 10^1^	4.5 × 10^3^
		Lake	2	1.2 × 10^3^	1.5 × 10^3^	4.9 × 10^2^	9.0	1.0 × 10^2^	2.3 × 10^3^
Aquatic plant	*Elodea nuttallii* (7), *Nuphar japonicum* (17), *Nymphoides peltata* (8), *Phragmites australis* (6), *Potamogeton berchtoldii* (4), *Potamogeton crispus* (5), *Potamogeton pusillus* (3)	River	18	1.5 × 10^3^	2.7 × 10^3^	5.8 × 10^2^	4.0	9.2 × 10^1^	9.2 × 10^3^
		Lake	32	2.1 × 10^2^	2.4 × 10^2^	1.4 × 10^2^	2.2	2.6 × 10^1^	1.3 × 10^3^
Snail	*Semisulcospira libertina* (50)	River	43	1.8 × 10^3^	4.1 × 10^3^	6.6 × 10^2^	3.6	2.1 × 10^1^	2.2 × 10^4^
		Lake	7	1.9 × 10^3^	1.5 × 10^3^	1.4 × 10^3^	2.5	3.8 × 10^2^	4.7 × 10^3^
Shrimp	*Paratya improvisa* (44)*, Palaemon paucidens* (24)*, Neocaridina* sp.(14)	River	106	1.2 × 10^3^	7.5 × 10^2^	9.9 × 10^2^	2.2	7.1 × 10^1^	3.2 × 10^3^
		Lake	1	2.0 × 10^3^					
Crayfish	*Procambarus clarkii* (53)*, Pacifastacus leniusculus* (20)	River	53	1.3 × 10^3^	1.1 × 10^3^	9.7 × 10^2^	2.4	9.7 × 10^1^	5.0 × 10^3^
		Lake	20	1.9 × 10^3^	6.9 × 10^2^	1.8 × 10^3^	1.4	9.3 × 10^2^	3.5 × 10^3^
Crab	*Eriocheir japonica* (55), *Geothelphusa dehaani* (2)	River	57	1.4 × 10^3^	1.1 × 10^3^	1.0 × 10^3^	2.4	6.6 × 10^1^	7.0 × 10^3^
Detritivore insect	*Stenopsyche marmorata* (95), *Isonychia japonica* (9), *Ephemera strigata* (7), *Drunella cryptomeria* (2)	River	125	2.9 × 10^3^	2.2 × 10^3^	2.2 × 10^3^	2.2	2.4 × 10^2^	1.4 × 10^4^
Carnivore insect	*Protohermes grandis* (44), *Parachauliodes continentalis* (12), *Macromia amphigena amphigena* (19), *Sieboldius albardae* (7), *Anotogaster sieboldii* (6), *Kamimuria tibialis* (6)	River	176	7.8 × 10^2^	8.1 × 10^2^	5.5 × 10^2^	2.3	6.9 × 10^1^	6.5 × 10^3^
		Lake	3	5.7 × 10^2^	1.1 × 10^2^	5.6 × 10^3^	1.2	4.8 × 10^2^	7.0 × 10^2^
Tadpole	*Rana catesbeiana* (9), *Lithobates catesbeianus* (3)	River	49	5.7 × 10^3^	4.0 × 10^3^	4.3 × 10^3^	2.3	5.0 × 10^3^	2.1 × 10^4^
		Lake	3	7.2 × 103	4.6 × 103	5.6 × 103	2.6	1.8 × 103	1.0 × 104
Adult amphibian	*Cynops pyrrhogaster* (22), *Rana rugosa* (16), *Rana ornativentris* (4), *Rana porosa porosa* (4), *Rana japonica* (3)	River	72	1.4 × 103	4.6 × 103	5.5 × 102	3.0	5.4 × 101	3.6 × 104
		Lake	12	8.9 × 102	5.9 × 102	7.2 × 102	2.0	1.9 × 102	2.2 × 103

## 2 Experimental design, materials and methods

### 2.1 The MOE monitoring

The MOE monitoring data were obtained for ^137^Cs activity concentrations for freshwater fish and aquatic organisms at multiple sites in Fukushima Prefecture since 2011, after the Fukushima Daiichi Nuclear Power Plant accident. The MOE monitoring dataset presented the following advantages: low ^137^Cs activity in water was quantified without it going below the detection limit; a large number of concentration factor (CF) values of fish and other aquatic organisms can be evaluated in a wide geographical range in Fukushima; and the water quality was measured in detail at every sampling location. Aquatic organisms included not only fish but also litter, plankton, periphyton, aquatic plants, aquatic insects, crustaceans, mollusks, and amphibians. From the calculation of the ^137^Cs concentration factors, it is evident that equilibrium between the biota and the water samples was not attained immediately after the accident. Because the data indicated the CFs were relatively stable after 2013, we calculated the CFs of ^137^Cs for the years 2013–2017.

### 2.2 Study area

The monitoring was conducted in five rivers (Uda River, Mano River, Niida River, Ota River, and Abukuma River) and three lakes (Lake Hayama, Lake Akimoto, and Lake Inawashiro) of Fukushima, Japan (Fig. 1, Fig. 2). Table 1 shows the geographic coordinates (latitudes and longitudes) of the sampling locations. The Abukuma River in central Fukushima flows through areas with relatively low contamination levels. The Abukuma river includes the monitoring sites Abukuma A and B. Lakes Akimoto and Inawashiro are located in central Fukushima, and Lake Hayama is a dammed lake upstream of the Mano River. Fish, aquatic organisms, litter, and water samples were collected at 2–4 monitoring sites per river or lake.

### 2.3 Sample collection and preparation

The sampling was conducted four times a year (spring: May to July, summer: August to September, autumn: October to November, and winter: December) at each site. Water was sampled at each monitoring site. Each water sample was filtered through a plankton net (72–75 μm mesh) to exclude organic and inorganic contaminants, the filtration process could not distinguish dissolved and particulate forms of ^137^Cs in water. The filtered water sample was used to measure pH, biochemical oxygen demand (BOD), chemical oxygen demand (COD), dissolved oxygen (DO), electric conductivity (EC), salinity, total organic carbon (TOC), suspended solid concentration (SS), turbidity and water temperature. The level of ^137^Cs activity in the water samples was determined by the ammonium phosphomolybdate method [3].

Freshwater fish and other aquatic organisms were sampled and ^137^Cs activity concentrations measured as whole-body wet samples. All biota samples were rinsed with water, chopped, homogenized, and frozen from −25 to −30 °C in plastic containers (U8; diameter = 50 mm; height = 62 mm). Detailed descriptions of sample collection and preparation for germanium gamma-ray spectrometer analysis are given in Ishii et al. (2019) [1].

### 2.4 Gamma spectrometric analysis

The ^137^Cs activity concentrations were measured using coaxial germanium detectors (Canberra GC2020 [Mirion Technologies, San Ramon, CA, USA], Canberra GC4020 [Mirion Technologies], ORTEC GMX 60–83 [Ametek Ortec, Oak Ridge, TN, USA], and GEM 40–76 [Ametek Ortec]) and well-type germanium detectors (Canberra GCW2523 [Mirion Technologies] and ORTEC GWL–90–15–XLB–AWT [Ametek Ortec]). The relative efficiency of these germanium detectors ranged from 23 to 60%. The instruments were calibrated using the standard volume radioactivity source for U8 containers (1 mm, 3 mm, 5 mm, 10 mm, 20 mm, 30 mm, and 50 mm) [Eckert & Ziegler, Berlin, Germany] and for Teflon tube containers (2 mm, 5 mm, 10 mm, 20 mm, and 40 mm) [Eckert & Ziegler]. Samples were measured considering <10% errors per net area counts. The counting time was extended up to 80,000 s. Gamma Station (Seiko EG&G Co. Ltd., Chuo-ku, Tokyo, Japan) and Gamma Explorer (Mirion Technologies) were used to analyze the γ-ray spectra for the 661.6382 keV of ^137^Cs. ^137^Cs activity concentration of samples was corrected for radioactive decay since the sample collection date.

### 2.5 Calculation of ^137^Cs CF for fish species

For the calculation of ^137^Cs CFs, the ^137^Cs activity levels of the freshwater fish were divided by the water ^137^Cs activity. The water ^137^Cs activity, and other water chemistry values which were measured simultaneously at the same monitoring site as fish collection were referenced from the monitoring data. When the values of BOD were described as < 0.5 in the monitoring data, they were replaced by 0.25, and when the values of SS were described as < 1, they were replaced by 0.5. If the sample was collected from multiple sampling points, the water ^137^Cs activity, and the water quality values of the sampling points were averaged. The CF values are summarized in Table 2, including 30 fish species (N = 1246). The dataset which associated CF values with ^137^Cs activity concentration of water and water chemistries are attached as a supplementary file to this article (Appendix A). The dataset variables are as follows: site_code: monitoring site code (see Table 1), site: monitoring site, ecosystem: the type of ecosystem (river or lake), samplingpoint: sampling point (see Table 1), date: date of sampling, season: season of sampling (spring, summer, autumn, winter), order: order of sample, family: family of sample, species: species of sample, habitat: habitat of fish (pelagic, benthopelagic, or demersal fish; based on fishbase.org information), N: number of merged fish individuals for Gamma spectrometric analysis, weight: weight of merged fish sample (kg), meansize: mean size of fish calculated by dividing the weight by N (g), remove_IO: removal of internal organs (1: remove, 0: not removed, na: unknown), Cs137: ^137^Cs activity concentration of fish (Bq kg^−1^), Cs137.w: ^137^Cs activity concentration of water (Bq kg^−1^), CF: ^137^Cs concentration factor (L kg^−1^), pH: pH of water, BOD: biochemical oxygen demand of water (mgL^−1^), COD: chemical oxygen demand of water (mgL^−1^), DO: dissolved oxygen of water (mgL^−1^), EC: electric conductivity of water (mS/m), salinity: salinity of water (psu), TOC: total organic carbon of water (mgL^−1^), SS: suspended solid concentration of water (mgL^−1^), turbidity: turbidity of water, and temperature: temperature of water (°C).

### 2.6 Calculation of ^137^Cs concentration factor for other aquatic organisms

Litter, plankton, periphyton, aquatic plants, aquatic insects, crustaceans, mollusks, and amphibians were obtained from the same monitoring sites as the fish. The ^137^Cs concentration factor was calculated in the same way as for freshwater fish. The aquatic organisms, their dominant species, and their CF values are summarized in Table 3. The dataset which associated CF values with ^137^Cs activity concentration of water and water chemistries are attached as a supplementary file to this article (Appendix B). The dataset variables are as follows: site_code: monitoring site code (see Table 1), site: monitoring site, ecosystem: the type of ecosystem (river or lake), samplingpoint: sampling point (see Table 1), date: date of sampling, season: season of sampling (spring, summer, autumn, winter), category1: category of aquatic organisms (litter, primary producer, mollusk, crustacean, aquatic insect, amphibian), category2: detailed category of aquatic organisms (litter, plankton, periphyton, aquatic plant, snail, shrimp, crab, crayfish, detritivore insect, carnivore insect, adult amphibian, tadpole), order: order of sample, species: species of sample, N: number of merged individuals for Gamma spectrometric analysis, weight: weight of merged sample, Cs137: ^137^Cs activity concentration of sample (Bq kg^−1^), Cs137.w: ^137^Cs activity concentration of water (Bq kg^−1^), CF: ^137^Cs concentration factor (L kg^−1^), pH: pH of water, BOD: biochemical oxygen demand of water (mgL^−1^), COD: chemical oxygen demand of water (mgL^−1^), DO: dissolved oxygen of water (mgL^−1^), EC: electric conductivity of water (mS/m), salinity: salinity of water (psu), TOC: total organic carbon of water (mgL^−1^), SS: suspended solid concentration of water (mgL^−1^), turbidity: turbidity of water, and temp: temperature of water (°C).

## Appendix A Supplementary data

Multimedia component 1 Multimedia component 1

Multimedia component 2 ^137^Cs activity concentration and concentration factors of freshwater fish in an EXCEL spreadsheet. Multimedia component 2

Multimedia component 3 ^137^Cs activity concentration and concentration factors of other freshwater aquatic organisms in an EXCEL spreadsheet. Multimedia component 3

## References

[1] Ishii Y., Matsuzaki S.S., Hayashi S. (2019). Different factors determine ^137^Cs concentration factors of freshwater fish and aquatic organisms in lake and river ecosystems. J. Environ. Radioact..

[2] Ministry of Environment Radioactive material monitoring Surveys of the water environment. http://www.env.go.jp/en/water/rmms/surveys.html.

[3] Hirose K., Aoyama M., Igarashi Y., Komura K. (2008). Improvement of 137Cs analysis in small volume seawater samples using the Ogoya underground facility. J. Radioanal. Nucl. Chem..

